# Local atomic structure of thin and ultrathin films *via* rapid high-energy X-ray total scattering at grazing incidence

**DOI:** 10.1107/S2052252519000514

**Published:** 2019-02-21

**Authors:** Ann-Christin Dippel, Martin Roelsgaard, Ulrich Boettger, Theodor Schneller, Olof Gutowski, Uta Ruett

**Affiliations:** a Deutsches Elektronen-Synchrotron DESY, Notkestraße 85, 22607 Hamburg, Germany; b Center for Materials Crystallography, Department of Chemistry, Aarhus University, Langelandsgade 140, 8000 Aarhus C, Denmark; c Institute for Materials in Electrical Engineering (IWE-2), RWTH Aachen University, Sommerfeldstraße 24, 52074 Aachen, Germany; d Advanced Photon Source, Argonne National Laboratory, 9700 S. Cass Avenue, Argonne, IL 60439, USA

**Keywords:** thin films, pair distribution function, high-energy X-ray diffraction, grazing-incidence diffraction, materials science, nanoscience, inorganic materials, nanostructures

## Abstract

Atomic pair distribution function (PDF) analysis is the most powerful technique to study the structure of condensed matter on the length scale from short- to long-range order. Today, the PDF approach is an integral part of research on amorphous, nanocrystalline and disordered materials from bulk to nanoparticle size. Thin films, however, demand specific experimental strategies for enhanced surface sensitivity and sophisticated data treatment to obtain high-quality PDF data. The approach described here is based on the surface high-energy X-ray diffraction technique applying photon energies above 60 keV at grazing incidence. In this way, reliable PDFs were extracted from films of thicknesses down to a few nanometres. Compared with recently published reports on thin-film PDF analysis from both transmission and grazing-incidence geometries, this work brought the minimum detectable film thickness down by about a factor of ten. Depending on the scattering power of the sample, the data acquisition on such ultrathin films can be completed within fractions of a second. Hence, the rapid-acquisition grazing-incidence PDF method is a major advancement in thin-film technology that opens unprecedented possibilities for *in situ* and *operando* PDF studies in complex sample environments. By uncovering how the structure of a layered material on a substrate evolves and transforms in terms of local and average ordering, this technique offers new opportunities for understanding processes such as nucleation, growth, morphology evolution, crystallization and the related kinetics on the atomic level and in real time.

## Abstract 2

The short- and long-range order of thin films with thicknesses down to 3 nm were studied by applying PDF analysis to data collected by surface high-energy X-ray diffraction at a time resolution on the scale of seconds.

## 1. Introduction   

Over the past two decades, materials scientists have gradually embraced the emerging potential of the atomic pair distribution function (PDF), in particular to describe the short-range order of amorphous and nanocrystalline phases, as well as local deviations from the average structure in periodic systems (Billinge & Kanatzidis, 2004 ▸; Billinge & Levin, 2007 ▸; Young & Goodwin, 2011 ▸; Playford *et al.*, 2014 ▸; Mancini & Malavasi, 2015 ▸; Keen & Goodwin, 2015 ▸). The PDF is essentially a histogram of the existing interatomic distances *r* represented in real space, regardless of the overall degree of ordering and the chemical and physical nature of the probed matter. Its growing importance originates in the increasing availability of instrumentation suitable to generate PDF data in the laboratory and at large-scale facilities. The combination of high-flux high-energy X-rays with large and fast area detectors, referred to as rapid-acquisition PDF (Chupas *et al.*, 2003 ▸, 2007 ▸), has especially boosted time-resolved PDF studies and is nowadays available at more and more synchrotron beamlines. At the same time, the advances in software for data treatment and modeling that make use of the ever expanding computational capabilities have equally contributed to the success of PDF analysis. In this work, we solely concentrate on the total scattering technique to obtain the atomic PDF (Egami & Billinge, 2013 ▸) and entirely omit the X-ray absorption spectroscopy based way to derive the related radial distribution function (Rehr & Albers, 2000 ▸).

A total scattering measurement is a diffraction measurement under specific conditions using X-rays, neutrons or, more recently, electrons (Abeykoon *et al.*, 2015 ▸). The collected scattering pattern in terms of intensity *versus* momentum transfer *Q* is converted into the reduced structure function *F(Q)* and then Fourier transformed into the reduced pair distribution function *G*(*r*) as described in great detail in the work by Egami & Billinge (2013 ▸). The term ‘total scattering’ refers to the fact that the entire pattern, *i.e.* the Bragg peaks (if any are present) as well as the diffuse scattering in between, is Fourier transformed and analyzed in real space. This constitutes a significant difference from the analysis of powder diffraction data in reciprocal space such as Rietveld refinement where the intensity in between the Bragg reflections is discarded as background. In total scattering, corrections are performed to isolate only the coherent scattering of the sample (Egami & Billinge, 2013 ▸). Here, the term ‘background’ rather denotes all scattering signals that stem from anything other than the sample, *e.g.* the sample container and surrounding atmosphere. Consequently, this background scattering must be removed prior to the Fourier transform for the PDF to purely represent the structure of the sample. This is achieved by carrying out a separate measurement of the sample environment without the sample, and subsequent subtraction of the scaled scattering pattern of the background from the actual sample data including background. As-derived PDFs can reveal correlations over a wide length scale in direct space, from the nearest neighbors in the range of a few ångströms up to coherent domains of several nanometres, *e.g.* crystallites or nanoparticles.

In order to obtain high-quality PDF data, it is essential to collect the scattering signal up to high momentum transfer *Q* = 4π sin θ/λ in the range of 20 to 30 Å^−1^, where λ is the wavelength and θ is the diffraction angle from Bragg’s law of diffraction. The wavelength is reciprocal to the photon energy *E* as *E* = *hc*/λ, with Planck’s constant *h* and the speed of light in vacuum *c*. The maximum obtainable momentum transfer *Q*_max_ is the upper integration limit used in the Fourier transform into the *G*(*r*) function and, hence, determines the real-space resolution (Proffen *et al.*, 2003 ▸). Since *Q* and λ are inversely proportional as noted above, it is obvious that *Q*_max_ is generally restricted by the wavelength. When an area detector is used, *Q*_max_ is geometrically limited to forward scattering up to 2θ of about 60° in practice. In order to have access to the required range in reciprocal space, the total scattering measurement is therefore most efficiently carried out using X-rays of high photon energies ≥ 60 keV (corresponding to λ ≤ 0.21 Å) provided by a synchrotron light source. In this case, a large area detector positioned at a short sample-to-detector distance readily covers a *Q* range ≥ 20 Å^−1^ simultaneously in a single exposure, generally enabling time-resolved PDF experiments. Nonetheless, dedicated laboratory instruments using Mo or Ag X-ray tubes with photon energies around 17.5 and 22.2 keV [wavelengths 0.71 and 0.56 Å (Thompson *et al.*, 2009 ▸)], respectively, and scanning point or linear detectors are playing an increasingly important role in PDF analysis. However, we must consider that the practical limit *Q*_max_ < 20 Å^−1^ in the case of Mo radiation or sources with even longer wavelengths inherently degrades the resolution *r* as described above. In addition, as the X-ray atomic form factor falls off exponentially with increasing *Q*, good counting statistics at high *Q*, where the intensities are low, are key to extracting reliable information on the short-range order scale in real space. When using an X-ray tube with a comparatively low incidence flux and long wavelength, the scattering signal can only be amplified by long counting times, in particular, at scattering angles of 2θ > 90°. In general, laboratory PDF measurements are useful when long data collection times are acceptable and the requirements on the real-space resolution are moderate.

In the structural study of thin layers and interfaces, surface X-ray diffraction (SXRD) under grazing incidence is a well established technique for crystalline films, especially under *operando* and *in situ* conditions, [compare, for example, Vlieg *et al.* (1988 ▸); Feidenhans’l (1989 ▸); Robinson & Tweet (1992 ▸); Fuoss *et al.* (1992 ▸); Ferguson *et al.* (2009 ▸); Richard *et al.* (2010 ▸)]. Recently, Gustafson *et al.* (2014 ▸) highlighted how SXRD using high-energy X-rays (which compress the diffraction pattern into a small angular range according to Bragg’s law) and a large and fast area detector revolutionized time-resolved structure determination of well ordered surfaces and epitaxially grown layers. In the case of amorphous thin films, however, the structural analysis in reciprocal space is much less straightforward; they lack periodic ordering, and hence their diffraction patterns are dominated by diffuse scattering, whereas distinct Bragg scattering, like crystal truncation rods or Bragg peaks, is absent. This is a typical situation in which PDF analysis is the superior method to derive structural information. While the PDF approach is commonly used in the study of bulk materials, its application to thin films is not as straightforward and can be challenging with respect to sample properties and instrumental demands, as will be discussed below. Here, we introduce rapid acquisition PDF analysis based on high-energy SXRD measurements, which represents a significant expansion of methods with respect to sensitivity and high-speed detection to determine the local structure of thin films. Now that this technique is available, it provides a new resource for insight into the structure of amorphous, disordered and polycrystalline thin films under real conditions in real time during their growth and operation with a potentially large impact for technologies including energy harvesting and storage, health, and smart electronic devices and appliances.

High-energy photons have the inherent property of large penetration power, even for materials with a high atomic number (*Z*). While this is an excellent precondition, *e.g.* for *in situ* experiments carried out in complex sample environments, it does not necessarily provide for favorable conditions for the study of samples with dimensions in the micrometre and nanometre range. In this case, the small sample size essentially limits the scattering signal rather than the absorption. This also holds true for thin films in which one dimension is commonly confined to below 1 µm, while the other two dimensions are typically beyond the millimetre scale. Aside from the low absolute amount of sample in a thin film, two major aspects have impeded PDF analysis of thin films: (i) the strong scattering from the substrate the film is deposited on, whose thickness exceeds the film thickness usually by at least three orders of magnitude, causing an unfavorable signal-to-background ratio, and (ii) texture, owing to different growth behavior parallel and perpendicular to the surface and/or in different crystallographic directions. Different approaches have been taken to circumvent these problems, including exfoliating the film off the substrate and grinding it (Bauers *et al.*, 2015 ▸; Kurzman *et al.*, 2015 ▸) as well as measuring the film on the substrate in transmission and carefully subtracting the dominant background signal of the amorphous substrate (Jensen *et al.*, 2015 ▸; Nakamura *et al.*, 2017 ▸; Shi *et al.*, 2017 ▸; Wood *et al.*, 2017 ▸). Both these methods have drawbacks: in the first case, structural changes may occur during the mechanical treatment and, in addition, any texture information is lost completely, whereas in the latter case, the signal-to-background ratio effectively sets a detection limit on the film thickness. Grazing-incidence (GI) geometry has been applied in thin-film and surface PDF studies using hard X-rays generated by synchrotron (Fischer-Colbrie *et al.*, 1988 ▸; Vaknin *et al.*, 2008 ▸; Stone *et al.*, 2016 ▸; Shyam *et al.*, 2016 ▸) or laboratory sources requiring long counting times (Eguchi *et al.*, 2010 ▸; Elschner *et al.*, 2011 ▸). The investigated layers exhibited minimum thicknesses of at least 25 nm to > 1 µm. In all these studies the diffraction data were collected at X-ray energies between 8 and 23 keV by scanning a small detector over a large 2θ range. By using high-energy X-rays of 100 keV for diffraction under grazing incidence and a large, fast area detector, we overcame the limitations of both the partially poor data quality owing to small *Q*_max_ and long data acquisition times. In this work, we present how we extracted and analyzed grazing-incidence pair distribution functions (GIPDFs) from films as thin as 3 nm at a time resolution down to 0.5 s, with the data collected by surface high-energy X-ray diffraction on a large area detector.

## 2. Experimental   

### 2.1. Sample preparation   

Metal and oxide thin films were investigated which were prepared by different deposition methods. In all cases, fused silica wafers (UniversityWafer, Inc., South Boston, USA) were used as substrates, providing a reproducible and, thus, easily scalable background signal due to its amorphous structure (Jensen *et al.*, 2015 ▸). We sputter-deposited platinum ultrathin films in an RF magnetron sputtering chamber specially designed for *in situ* PDF measurements (Roelsgaard *et al.*, 2019 ▸). In the example described below, the Pt was sputtered for 30 s at an RF power of 12 W in pure Ar plasma at a pressure of 5 × 10^−2^ mbar (1 mbar = 100 Pa) and around 30°C substrate temperature. The highly textured [111] film (discussed in Section 3.4) was fabricated by the standard process for Pt electrodes described by Schneller & Waser (2007 ▸). HfO_2_ and ZrO_2_ thin films were prepared by the chemical solution deposition (CSD) routes described by Starschich and coworkers (Starschich *et al.*, 2015 ▸; Starschich & Böttger, 2018 ▸). We varied the film thickness by repeating the spin-on process 1–14 times to produce films of 15–200 nm thickness. The spin-coated samples were mostly heat-treated as described in the literature, *i.e.* the pre-annealing of the wet films was performed on a laboratory hotplate at 295°C and the crystallization was carried out in a rapid thermal annealing device at 800°C. For the ZrO_2_ sample with an annealing temperature of 900°C, the thermal treatment differed as it was applied during an *in situ* X-ray total scattering measurement. For this purpose, a pre-annealed ZrO_2_ sample was heated on a silicon nitride hot plate (Bach Resistor Ceramics GmbH, Werneuchen, Germany) at 10°C min^−1^ in air to the final temperature of 900°C.

### 2.2. Data acquisition and treatment   

**Figure 1:**
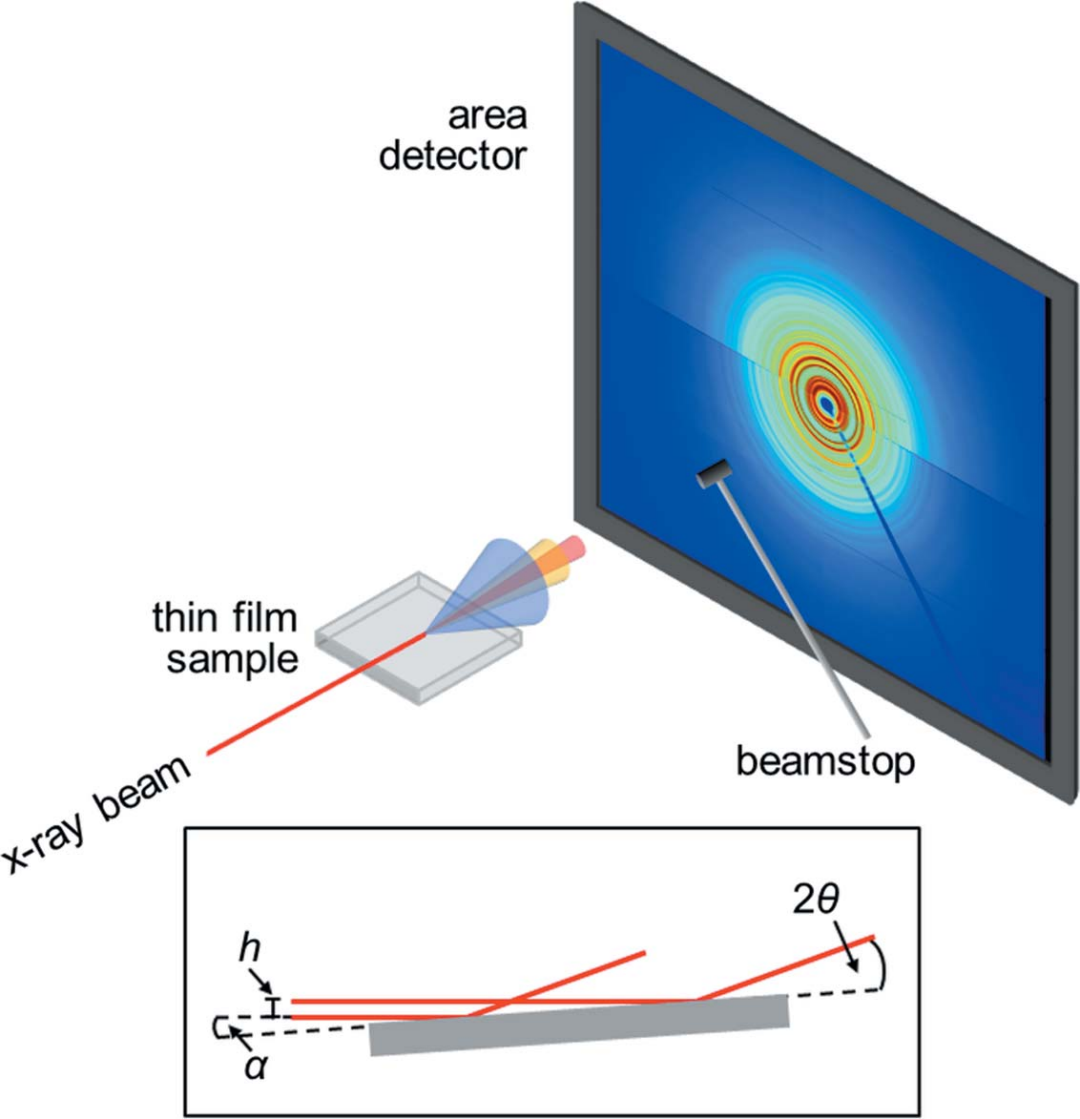
Schematic illustration of the grazing-incidence geometry (all optical components from the undulator source up to the pinhole in front of the sample have been omitted for clarity). In the inset drawing, the beam height *h*, the fixed incidence angle α and the varying diffraction angle 2θ are defined. In the 2D diffraction pattern, the so-called sample horizon divides the image into an upper and a lower half. In the lower half, the intensities are less than in the upper part as the diffracted signal is partly absorbed by the substrate it passes on its way to the detector, whereas the upward scattering reaches the detector unattenuated.

The high-energy surface X-ray diffraction experiments were performed in the second experimental hutch of Beamline P07 at PETRA III, DESY, Hamburg, Germany (Gustafson *et al.*, 2014 ▸). A schematic of the experimental setup is given in Fig. 1 ▸. 1D silicon compound refractive lenses (Bertram *et al.*, 2016 ▸) were used to focus X-rays of 98.3 keV photon energy to a beam size of around 3 × 500 µm^2^ (vertical times horizontal in terms of full width at half maximum, FWHM). The films were aligned parallel to the direct beam in height and tilt angles on the surface diffractometer and measured under incidence angles of the order of 0.03–0.04°. Diffraction data were collected on a PerkinElmer XRD1621 flat panel detector positioned at a sample-to-detector distance (SDD) of 395 mm. In order to calibrate the SDD and wavelength, a capillary with LaB_6_ powder was measured as a standard sample. The instrumental resolution in grazing-incidence geometry was determined from systematic measurements of CeO_2_ powder dispersed over the surface of a fused silica substrate at various SDDs from 305 to 695 mm. Azimuthal integration of the 2D diffraction patterns and calibration were carried out using the *pyFAI* package (Kieffer & Wright, 2013 ▸), and subsequent transformation into the PDF was performed using *PDFgetX3* (Juhás *et al.*, 2013 ▸) implemented in the *xPDFsuite* software (Yang *et al.*, 2014 ▸). The applied *Q*_max_ values are listed in Table S1 of the supporting information. Structural refinements in real space were carried out in *PDFgui* (Farrow *et al.*, 2007 ▸).

## 3. Results and discussion   

### 3.1. Surface-enhanced sensitivity   

Compared with the thickness of a thin film in the nanometre regime, the height of the focused X-ray beam of approximately 3 µm FWHM is up to three orders of magnitude larger. While the penetration depth of photons in a material (Feidenhans’l, 1989 ▸) is energy dependent at incidence angles above the critical angle of total external reflection α_c_, it is independent of the photon energy below α_c_ (see Fig. S1 of the supporting information). Theoretically, the penetration depth calculates to a few nanometres for perfect satisfaction of the grazing-incidence condition below α_c_. As the increase of the penetration depth for incidence above the critical angle is very steep and scales with the photon energy, even minor deviations from the ideal total reflection geometry give rise to scattering from the substrate. As a consequence, this effect is particularly observable in high-energy GIPDF experiments since the relevant angles lie in the range of a few tens of millidegrees. Violations of the total reflection condition partly stem from sample properties such as roughness and non-flatness. In addition, the characteristics of the X-ray beam (full beam larger than the FWHM value, finite divergence and energy spread) add scattering from the substrate as well.

**Figure 2:**
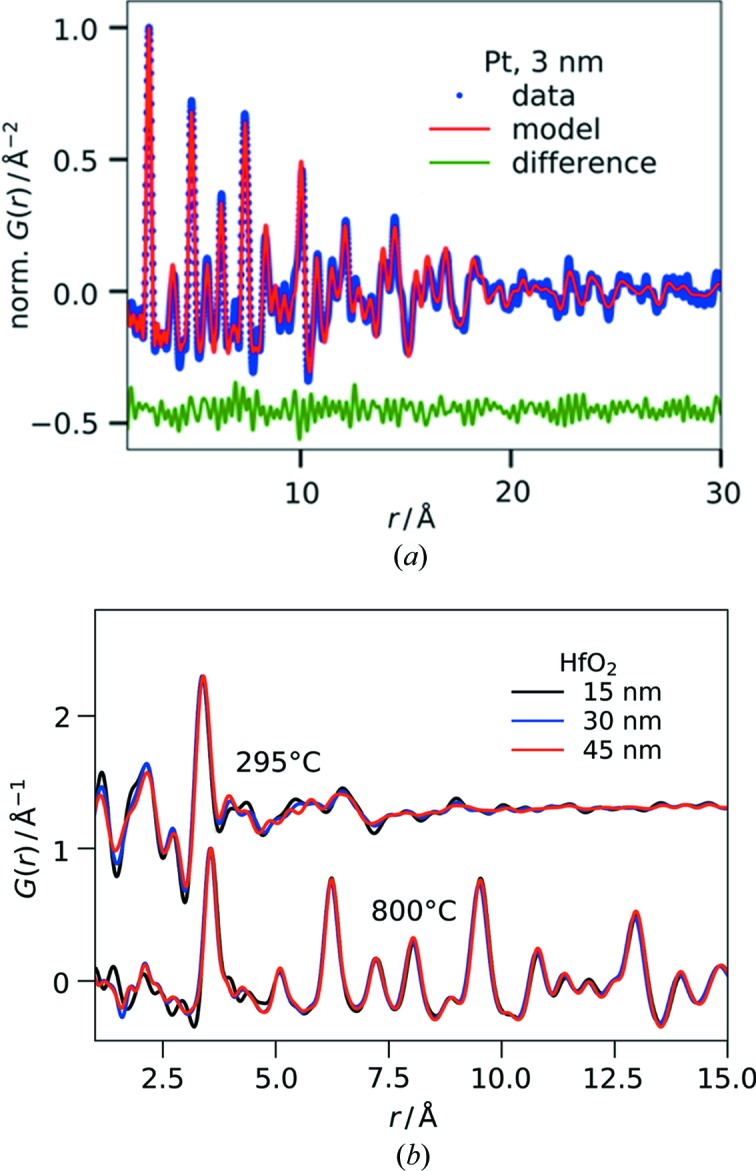
(*a*) PDF data and refinement of a 3 nm sputter-deposited Pt layer on fused silica; *R*_w_ = 24% for the *r* fitting range 1.75–60 Å (shown only to 30 Å for clarity); (*b*) PDFs of HfO_2_ thin films of different thicknesses on fused silica deposited by chemical solution deposition and heat-treated at the indicated temperatures [offset in *G*(*r*) for clarity]. The film thickness increases in steps owing to the preparation by stacking multiple coatings of 15 nm each, *i.e.* the samples correspond to 1, 2 and 3 coatings, respectively.

In the context of these considerations on signal-to-background ratio, we examined the detection limit of our GIPDF setup in relation to film thickness and sample composition. For this purpose, thin films of different materials and thicknesses were measured. Fig. 2 ▸(*a*) depicts the PDF and its structural fit of a sputter-deposited platinum layer on fused silica taken from an *in situ* sputtering data set (Roelsgaard *et al.*, 2019 ▸). A sequential refinement was carried out starting from the thickest film of the data set and going backwards in the series towards thinner samples. Under the assumption of a linear deposition rate and homogeneous coverage of the substrate, the extrapolated thickness of the selected sample is approximately 3 nm. When inspecting the residual given as the difference curve in Fig. 2 ▸(*a*), it mostly reflects random noise and no significant structural features are unaccounted for in the fitting. Hence, we consider the results from the refinements to be reliable structural information on the ultrathin Pt film. The obtained structural fit agrees with the data to *R*_w_ = 0.24 as expressed by the weighted *R* factor for the residual of the least-squares regression. This *R*_w_ value is comparable with thin-film PDF refinements in transmission geometry for layers around 100 times thicker (Jensen *et al.*, 2015 ▸).

As Pt is a high-*Z* material, it produces a strong scattering signal. We chose hafnium oxide thin films as an example for less strongly scattering materials. Fig. 2 ▸(*b*) shows the PDF data for three HfO_2_ films of 15–45 nm before and after crystallization, respectively, which are the thinnest samples in a series of 1–6 spin-coating steps (15–90 nm thickness). Fig. S2 illustrates the corresponding background scaling in reciprocal space performed before Fourier transformation for the two thinnest films and, for comparison, the thickest film. All PDFs extracted for the layers of ≥ 30 nm thickness were of essentially identical quality. Only the PDFs of a single coating exhibit a slightly higher noise level. While this does not essentially affect the high-intensity peaks at medium and high *r*, the PDF peak representing the nearest-neighbor Hf—O distance at 2.1 Å does not stand out as clearly as it does for the thicker samples. In principle, the low-*r* region is the most prone to inaccuracies because here the termination ripples from the Fourier transform are strongest. For materials such as HfO_2_ that are composed of elements with largely differing *Z* values, the bonds between the heavy elements (Hf—Hf) on the one hand and bonds between the light elements (O—O) or the heteroatomic bonds (Hf—O) on the other hand generate PDF peaks of largely varying intensity. Consequently, the high contrast between the strong and the weak peaks increases the challenge to resolve the Hf—O PDF peak at 2.1 Å of low intensity from the low-*r* noise, especially in the vicinity of the adjacent strong Hf—Hf peak at 3.5 Å. Nonetheless, the PDFs of the 15 nm layers still yield useful and reliable information on the local structure of the HfO_2_ films. It is noteworthy that the detection limit is similar for the crystalline and the disordered films heat-treated at 800 and 295°C, respectively. This observation is particularly remarkable when comparing the diffraction patterns of both sample types (see Fig. S2). In principle, the background subtraction for the crystalline samples with distinct, strong Bragg peaks is more straightforward and less error-prone than the separation of the broad, weaker scattering signal of the disordered films from that of the amorphous substrate. Indeed, the higher signal-to-background ratio of the data from the crystalline films allowed us to use higher *Q*_max_ values than for the disordered samples. Similarly, a decreased *Q*_max_ was applied when Fourier transforming the reduced scattering intensity from the thinnest film within the series of crystalline samples. Consequently, care has to be taken when quantitatively comparing the PDF peak widths in the two data series to disentangle the contributions from the samples themselves and the data treatment. Overall, these results demonstrate and emphasize that high data quality and careful data treatment are key to minimizing the detectable film thickness.

### 3.2. Instrumental broadening   

Generally, the experimental setup (sample dimensions, wavelength, sample-to-detector distance, pixel size of the area detector *etc.*) determines the angular resolution in reciprocal space. The limited *Q*-space resolution in turn affects the PDF by damping the *G*(*r*) function, *i.e.* reducing the PDF peak intensities with increasing *r* (Petkov, 2012 ▸; Olds *et al.*, 2018 ▸). This effect is accounted for by fitting the parameter *Q*_damp_ to data from a calibrant. In most modeling software, *Q*_damp_ is typically approximated as a Gaussian envelope to the PDF that reflects an assumed Gaussian peak shape in *Q* space. When the instrumental broadening in reciprocal space is *Q*-dependent, the PDF peaks vary in peak width with *r* [see Olds *et al.* (2018 ▸) and references therein]. *PDFgui*, which was used for modeling the presented data, applies a parameter *Q*_broad_ based on the approximation of linearly increasing Δ*r*/*r* and again under the assumption of Gaussian peak profiles.

**Figure 3:**
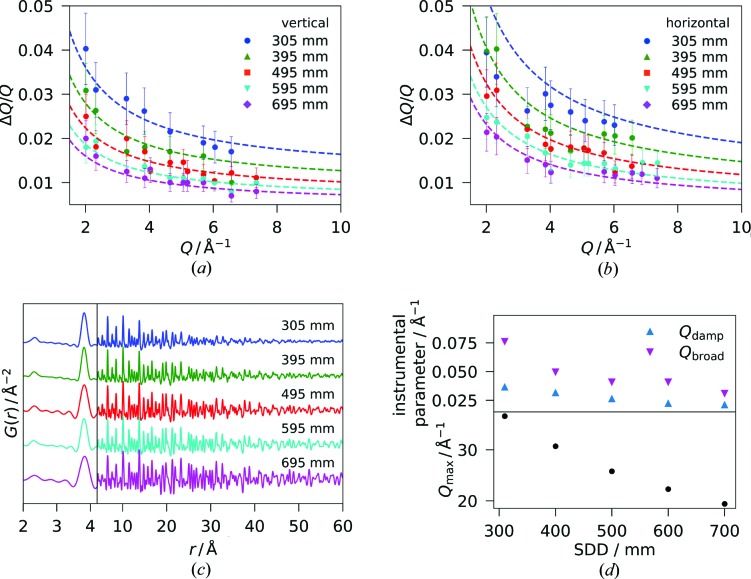
Instrumental resolution (dots: measured data; lines: modeled instrumental resolution function) at different sample-to-detector distances for the grazing-incidence geometry in (*a*) the vertical and (*b*) horizontal directions, obtained from measurements of CeO_2_ powder dispersed over a fused silica surface. Data were measured at a photon energy of ∼100 keV and recorded on a PerkinElmer XRD1621 detector. (*c*) Derived PDFs for the respective sample-to-detector distances [offset on *G*(*r*) axis, low-*r* range up to 4.2 Å magnified]. (*d*) Applied *Q*_max_ and instrumental parameters *Q*_damp_ and *Q*_broad_ from fits of the PDFs in (*c*) to the ICSD reference 72155 versus the distance between sample and detector.

Conventional PDF measurements on bulk samples in transmission geometry usually employ a beam size of the order of 0.2–1 mm^2^, which matches the sample dimensions. At the same time, a comparatively large beam ensures good statistical average in powder diffraction type measurement. In the grazing-incidence experiments applied in this work, however, the photon beam is focused into a horizontal line of 3 × 500 µm^2^. In this way, the incident flux on the film is optimized by limiting the length of the footprint, *i.e.* the irradiated area of the sample along the beam direction (compare Figs. 1 ▸ and S3a). In our high-energy GIPDF experiment performed with a beam height *h* of 3 µm, the incident angle α of 0.03–0.04° creates a footprint *f* = *h*/sin α of 4–5 mm length (Fig. S3b). The projected beam width on the detector is *Q*-dependent and amounts to ∼2 mm at *Q* ≃ 20 Å^−1^ (see Fig. S3c). Here, the applied high photon energy works in favor of only a moderate projection effect due to the compressed diffraction pattern (at 100 keV, *Q* in reciprocal ångströms and 2θ in degrees take very similar values, *e.g.* 20 Å^−1^ is equivalent to 23°). Figs. 3 ▸(*a*) and 3(*b*) depict the corresponding resolution in reciprocal space obtained for a flat sample of ceria powder, expressed in terms of Δ*Q/Q* for varying SDD and separately for the vertical and the horizontal directions. Clearly, the instrumental resolution is *Q* dependent, but obviously by nonlinear correlations that vary with the azimuthal angle. For this reason, the *Q*_broad_ calculation in *PDFgui* described above does not accurately model the peak broadening in *G*(*r*). In future GIPDF studies, it should be replaced by a more suitable description and implemented in more flexible modeling software such as *Diffpy-CMI* (Juhás *et al.*, 2015 ▸), but such a development is beyond the scope of this work. However, we presume that the linear approximation of *Q*_broad_ yields PDFs of sufficiently high validity for meaningful structural analysis considering that the deviation of the instrumental resolution from the linear approximation becomes rather small at moderate to high *Q*.

In order to put the obtained Δ*Q*/*Q* values into context, we do a simple comparison with the purely sample-related size broadening according to Scherrer’s formula (Langford & Wilson, 1978 ▸). When expressing the Scherrer equation as Δ*Q*_FWHM_ *=* 2π*K/D* with the shape factor *K* = 0.9 for spherical crystallites, it is evident that nanocrystallites of size *D* ≤ 5 nm, which are commonly studied by PDF analysis, cause a similar broadening Δ*Q* of the Bragg peaks as the grazing-incidence geometry.

Over the investigated SDD range from 305 to 695 mm, Δ*Q/Q* increases by more than double at the utilized photon energy of ∼100 keV. Varying the SDD not only affects the reciprocal-space resolution, but also the detectable *Q* range and, thus, the upper limit *Q*_max_ of the Fourier transform into the PDF. Fig. 3 ▸(*c*) illustrates the resulting PDFs, and Fig. 3 ▸(*d*) gives the corresponding values of *Q*_max_ and the instrumental parameters for damping *Q*_damp_ and peak broadening *Q*_broad_ from fittings in *PDFgui* of the fully azimuthally integrated data from the flat ceria powder sample. There is a consistent decreasing trend for *Q*_damp_ and *Q*_broad_ with increasing SDD so it may be expected that both the damping and the peak broadening effects diminish when the detector and the sample are moved further apart. This holds true only for the damping, as can be seen from the extent of correlations at large *r* that grows with increasing SDD. By contrast, the real-space resolution decreases with increasing SDD due to decreasing *Q*_max_, *i.e.* the FWHM of the PDF peaks nearly doubles over the given SDD range; for example, see the peak at 3.8 Å in Fig. 3 ▸(*c*). For our studies, we chose an SDD of 395 mm as the sweet spot with regard to resolution in real space.

### 3.3. Phase analysis   

**Figure 4:**
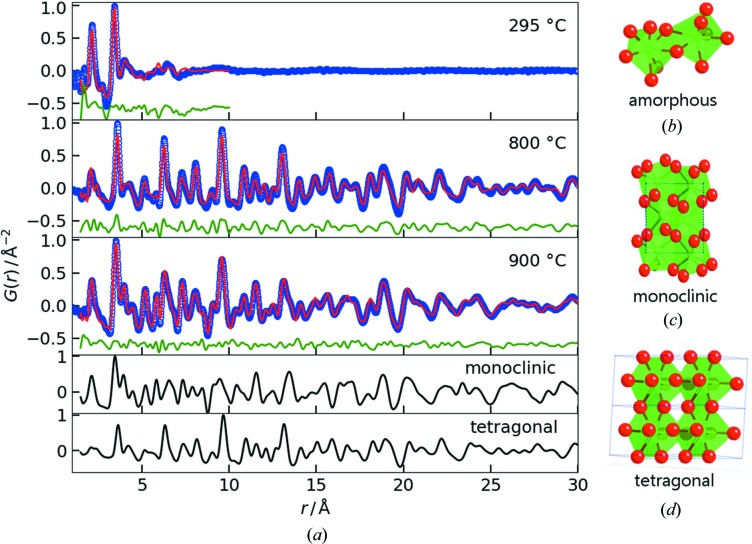
(*a*) (Top to bottom) PDFs with fits of a pre-annealed spin-coated ZrO_2_ film heated to 295°C, and two crystalline ZrO_2_ films prepared from equally pre-annealed layers and heated to 800°C in a rapid thermal annealing process with a heating rate of ∼100°C min^−1^, and 900°C in air at a slow heating rate of ∼10°C min^−1^, respectively (blue dots: data, red lines: calculated model, green line: difference curve, offset of −0.5 Å^−2^ for clarity), along with the calculated reference PDFs from the ICSD (references 658755 for monoclinic and 93124 for tetragonal with reduced values for *U*_iso_ extrapolated to room temperature). Structural models of (*b*) the double-polyhedron structural motif of the disordered amorphous phase (Tyrsted *et al.*, 2014 ▸), (*c*) the monoclinic and (*d*) the tetragonal structure; green and red spheres represent zirconium and oxygen ions, respectively.

For comparison of the data quality from our high-energy GIPDF data with conventional PDFs from nanoparticles, we performed phase analysis in real space on ZrO_2_ films of 200 nm thickness and heat-treated at different temperatures between 295 and 900°C. The results are presented in Fig. 4 ▸ along with the calculated PDFs of the references for the tetragonal and monoclinic phases [Inorganic Crystal Structure Database (ICSD) codes 93124 and 658755]. In the PDF of the film annealed at 295°C, two clear peaks at 2.1 and 3.4 Å are visible which correspond well with the nearest-neighbor Zr—O and Zr—Zr distances in the monoclinic structure. Beyond 4 Å, only broader features appear, indicating that the sample does not exhibit any long-range order. Fig. 4 ▸ (top) shows the corresponding data and the fit against the monoclinic phase in the *r* range 1.5–10 Å (*R*_w_ = 0.34). Attempts to fit the PDF against the tetragonal phase failed. *In situ* studies of the solvothermal synthesis of ZrO_2_-based nanoparticles (Tyrsted *et al.*, 2014 ▸; Dippel *et al.*, 2016 ▸) revealed comparable PDFs for an amorphous intermediate state modeled as a double-polyhedron structure with local monoclinic order. This building block was found to occur during the reaction from different starting materials and under varying reaction conditions. It is noteworthy that an intermediate which fits to the same structural short-range order is also found in this CSD route although yet again very different chemicals and processing parameters were employed. Hence, the ubiquity of the double-polyhedron structural element indicates its high stability in many chemical environments at moderately elevated temperatures.

Upon further heating to 800 and 900°C, as described in Section 2.1, the amorphous ZrO_2_ films crystallize. Visual comparison of the PDFs given in Fig. 4 ▸, in particular with respect to the intensities of the peaks at 6.3, 9.7 and 11.2 Å present in the tetragonal phase, suggests that the two samples comprise different phase ratios of the monoclinic (*m*) and tetragonal (*t*) phases. Fitting the data against the two reference phases and relating the two obtained scale factors yields a number ratio *t*:*m* of approximately 3:1 for the sample rapidly heated to 800°C and the opposite ratio *t*:*m* of approximately 1:3 for the layer slowly heated to 900°C.

Polymorphism is a known phenomenon in ZrO_2_, and numerous studies describe how to stabilize the high-temperature tetragonal phase in nanoparticles (Shukla & Seal, 2005 ▸). In the study of polymorphism of solvothermally synthesized ZrO_2_ nanoparticles (Dippel *et al.*, 2016 ▸), the *in situ* PDF data showed that chemical similarity of the amorphous intermediate and the final product governs the reaction kinetics. On the short-range order scale, the phase transformation from monoclinic to tetragonal generally requires that the shortest Zr—Zr distance increases from 3.5 to 3.6 Å (see Fig. S4) and that the coordination number increases from 7 to 8. For the nanoparticles, this transformation proved to be slow which determined the overall synthesis rate. Both of the crystalline thin-film samples studied in this work were obtained from the same amorphous solid with a monoclinic local structure like in the nanoparticle study, but the findings on the phase ratios are inconsistent with the earlier study. In the spin-coating process, the tetragonal portion of the films is large when the heating ramp is steep and the material is left with little time for structural rearrangement on the short-range order scale. By contrast, the nanoparticles require longer reaction times to form a higher ratio of tetragonal to monoclinic phase. However, the two synthesis methods differ too much in terms of applied thermal treatment, pressure, presence of solvent, sample dimensions *etc* to directly compare the outcomes. Hence, a systematic study is planned in order to determine the origin of the polymorphism in the thin-film route.

### 3.4. Texture   

**Figure 5:**
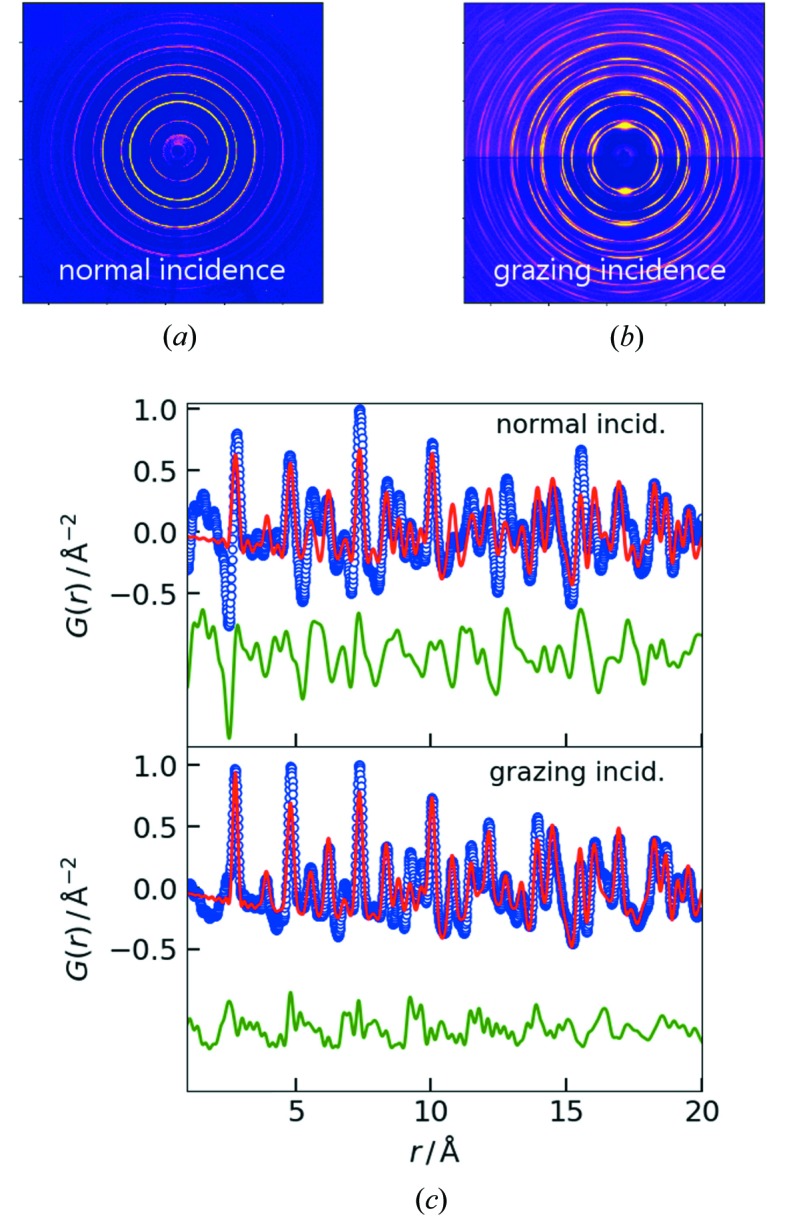
2D and background-subtracted X-ray diffraction patterns of a 50 nm thin film of Pt with [111] texture collected in (*a*) normal incidence (transmission) and (*b*) grazing-incidence geometry and (*c*) the corresponding PDFs with fits to the ICSD reference [database code 243678 (blue dots: data, red line: calculated PDFs from fit, green line at negative offset: difference between data and calculated PDFs)]. The background was subtracted by manually scaling the separately collected fused silica frame so as to eliminate the broad scattering features from the amorphous substrate at low *Q*, using the same background scale for the 2D and 1D data.

A varying degree of texture is a very common phenomenon in thin films. Between the extreme cases of single-crystal-like epitaxial and fully randomly oriented polycrystalline films, there are different degrees of texture. In a 2D diffraction pattern, texture shows as non-uniform intensity that depends on the azimuthal angle along a Debye–Scherrer ring. We investigated sputter-deposited Pt thin films that have a tendency to develop fiber texture, *i.e.* the close-packed {111} planes of the crystallites are preferentially oriented parallel to the substrate surface (out-of-plane direction along the film normal), whereas their orientation is randomly distributed around the film normal (in-plane direction). 2D diffraction patterns of a 50 nm Pt film with preferred [111] orientation are depicted in Fig. 5 ▸ and were recorded in transmission under normal incidence [Fig. 5 ▸(*a*)] and under grazing incidence [Fig. 5 ▸(*b*)]. The large differences between the diffraction patterns originate in the scattering vector preferentially probing the structure along the out-of-plane direction in grazing incidence and along the in-plane direction in normal incidence. Azimuthal integration of the diffraction data over 360° in the case of normal incidence and over the 180° of the upper half of the grazing-incidence data results in the PDFs displayed in Fig. 5 ▸(*c*). Both data sets are equivalent with respect to peak position and peak width which correspond well with the PDFs calculated for randomly oriented face-centered cubic Pt based on the reference data (ICSD code 243678). The relative peak intensities, however, vary significantly from the calculated PDF for the isotropic case in largely different ways for the two data sets. As a result, fits of both PDFs against the reference reveal varying systematic structural deviations from the powder average, including non-structure-related deep minima at low *r* in the transmission geometry case. It is evident that the conventional PDF analysis approach, which presumes random orientation, fails to quantitatively describe the structure of textured films and requires some mathematical input such as an orientation distribution function as proposed by Gong & Billinge, (2018 ▸). Such a disentanglement of structure and orientation would not only benefit thin-film PDF analysis, but also the real-space modeling of bulk materials with preferred orientation, *e.g.* natural fiber-textured materials such as bone or wood, and machined engineering materials like wires and rolled sheets.

## 4. Conclusions   

In this work, we demonstrate how high-energy surface X-ray diffraction in grazing-incidence geometry using a fast area detector is successfully applied in the study of the local and average structure of amorphous and crystalline thin films. Examples of different film materials and thicknesses illustrate the power and frontiers of the high-energy GIPDF technique. The small incidence angle of the X-ray beam creates a large footprint on the sample in the range of several millimetres which is projected onto the area detector. Nevertheless, the resulting reciprocal-space resolution was found to be similar to the broadening effects from small nanoparticles commonly studied by PDF. In comparison with the transmission geometry in which the X-ray beam travels through the entire substrate cross section, the surface sensitivity of the grazing-incidence method enormously enhances the signal-to-background ratio. Depending on the scattering power of the sample, layers of thicknesses down to 15 nm for oxides and 3 nm for metals gave powder-like diffraction patterns and reliable high-quality PDFs. In addition, the acquisition times were of the order of seconds or less. Thus, PDF analysis is now not only effectively applicable to thin films about ten times thinner than previously reported, it can also be performed with far better time resolution. In combination with the high penetration power of the high-energy X-rays, our GIPDF technique therefore provides favorable conditions for *in situ* studies of thin-film operation and growth in complex sample environments. Every technology that applies amorphous, disordered or polycrystalline films made from materials such as semiconductors, transparent conductive oxides or thermoelectrics will potentially benefit from these capabilities. Consequently, PDF analysis is henceforth expected to play a significant role in improving the efficiency of solar cells, displays, smart windows *etc*. Likewise, the resilience of passivation layers protecting against corrosion from humidity, bodily fluids, acid or other harsh environments, as well as the durability of hardening coatings for tools and dental materials may be enhanced based on GIPDF studies. Texture and preferred orientation in thin films alter the relative peak intensities of the derived PDFs, but leave the peak positions and widths unaffected. Hence, qualitative structural information such as bond lengths and crystallite size are available from the PDFs of textured films using isotropic modeling.

## Supplementary Material

Supporting figures and table. DOI: 10.1107/S2052252519000514/ro5016sup1.pdf
