# Supplementary material for: Local atomic structure of thin and ultrathin films via rapid high-energy X-ray total scattering at grazing incidence
Source: IUCrJ. 2019 Feb 21;6(Pt 2):290–8. doi: 10.1107/S2052252519000514 (PMC6400183; doi:10.1107/S2052252519000514)
Supplement: Supplementary file 1 [file m-06-00290-sup1.pdf]

IUCrJ

Volume 6 (2019)

Supporting information for article:

Local atomic structure of thin and ultrathin films *via* rapid high-energy X-ray total scattering at grazing incidence

Ann-Christin Dippel, Martin Roelsgaard, Ulrich Böettger, Theodor Schneller, Olof Gutowski and Uta Ruett

Supplementary information

Local atomic structure of thin and ultrathin films *via* rapid high-energy X-ray total scattering in grazing incidence

Ann-Christin Dippel[a], Martin Roelsgaard[b], Ulrich Böttger[c], Theodor Schneller[c], Olof Gutowski[a], Uta Ruett[d]

[a] *Deutsches Elektronen-Synchrotron DESY, Hamburg, Germany*
[b] *Center for Materials Crystallography, Department of Chemistry, Aarhus University, Denmark*
[c] *Institute for Materials in Electrical Engineering, RWTH Aachen University, Germany*
[d] *Advanced Photon Source, Argonne National Laboratory, 9700 S. Cass Avenue, Argonne, IL 60439, USA*

Table of contents

Figure S1 Penetration depth in dependence of photon energy

Figure S2 Background subtraction in reciprocal space for spin-coated HfO_2 thin films of different film thickness on fused silicon substrates

Figure S3 Illustration and calculated values of the footprint and its projected width on the area detector

Figure S4 Short-range order scale of ZrO_2 thin films and reference PDFs (magnified low-*r* region of Figure 4a)

Table S1 Q_{max} values for the PDFs shown in the main text

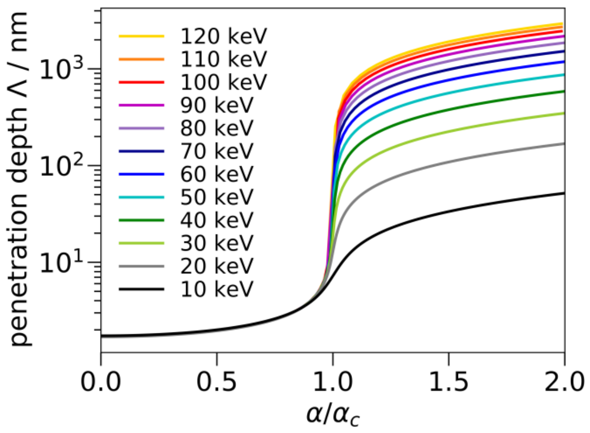

Figure S1: Penetration depth of photons of various energies calculated according to R. Feidenhans'l, (1989), Surf. Sci. Rep. 10, 105–188, exemplarily for Cu as a material without absorption edges within the illustrated energy range.

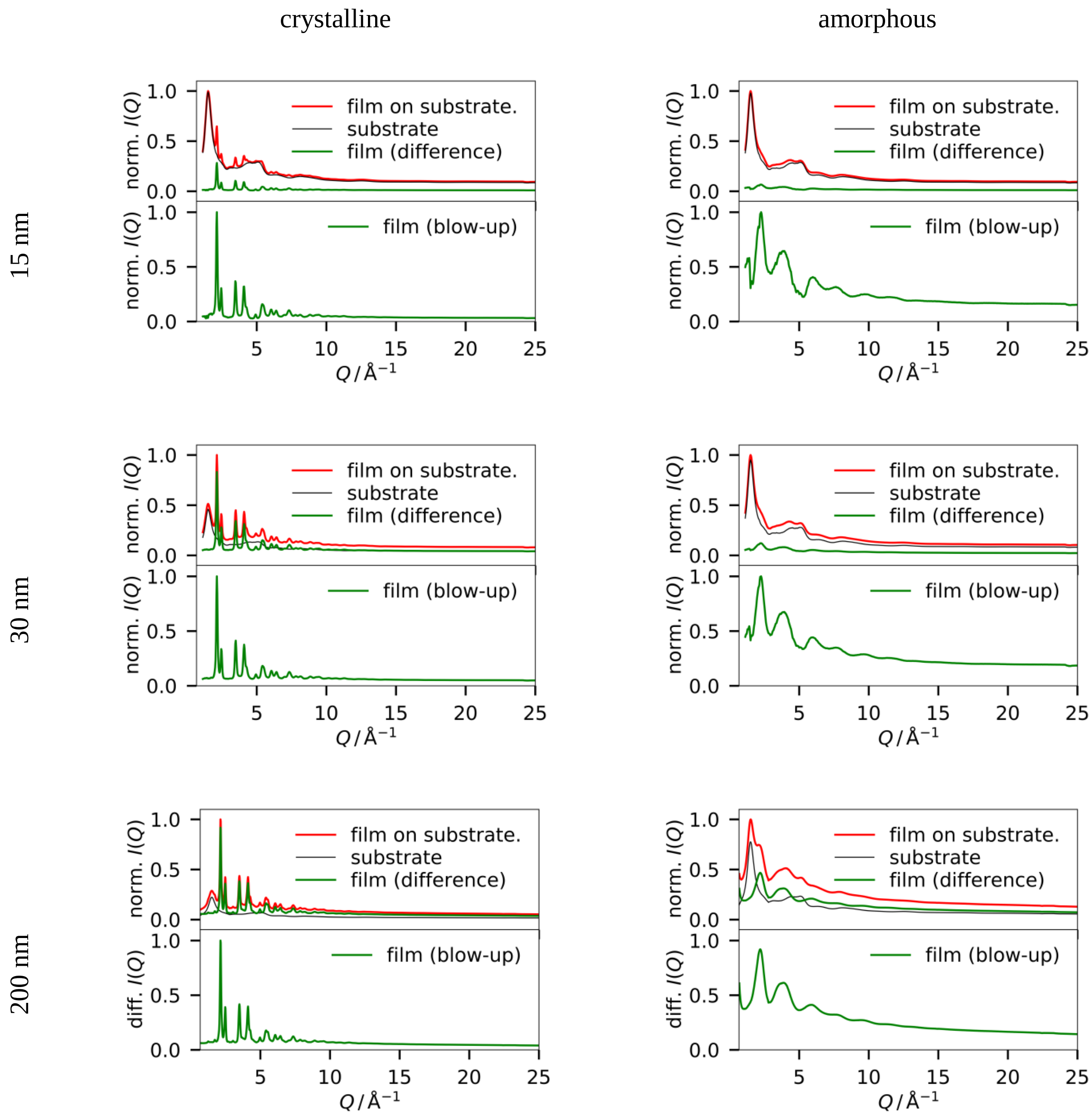

Figure S2: Integrated XRD patterns for HfO_2 layers on fused silica at different thicknesses, prepared by various repetitions of the coating process, and heat treated at 800 °C (crystalline) and 295 °C (amorphous). The plots show the integrated data of the samples and the scaled background patterns from the fused silica substrate, and the magnified subtracted signals for each case.

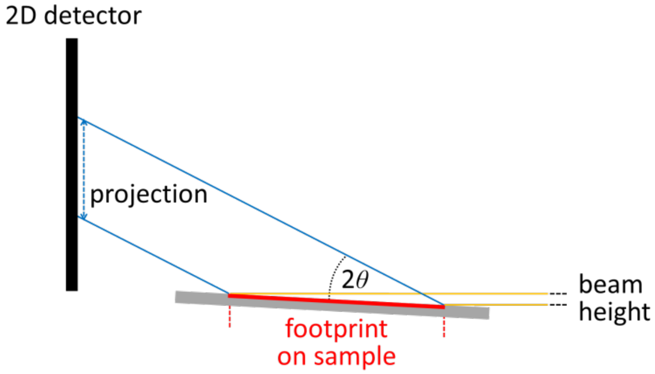

(a)

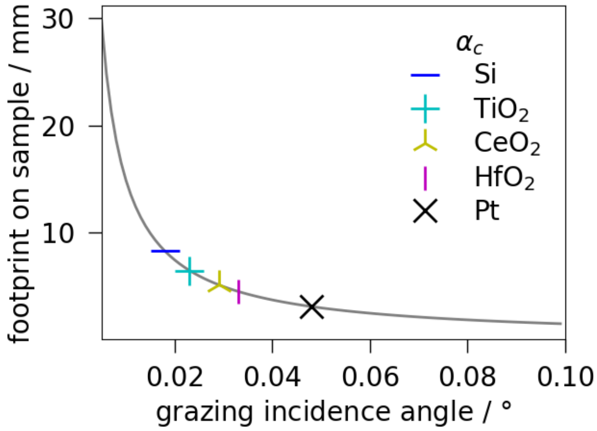

(b)

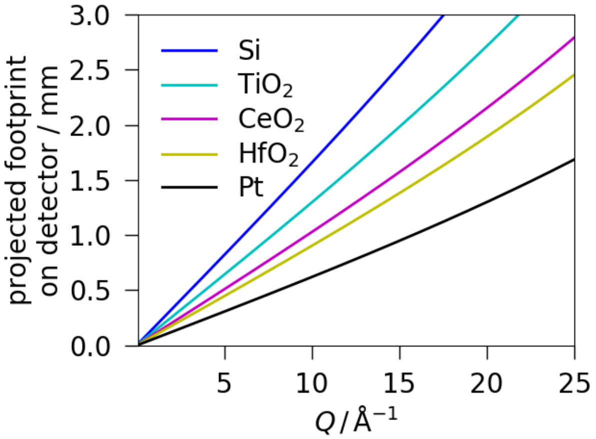

(c)

Figure S3: (a) Schematic illustration of the grazing incidence geometry (incidence angle exaggerated for clarity) and projection of the footprint on the area detector; (b) footprint in dependence on the incidence angle with marked critical angles for some selected materials; (c) projected footprint on the area detector calculated for the critical angles of the indicated materials; all values are calculated for a beam height of 3 μm based on simple trigonometric considerations for the vertical scattering plane and a photon energy of 100 keV.

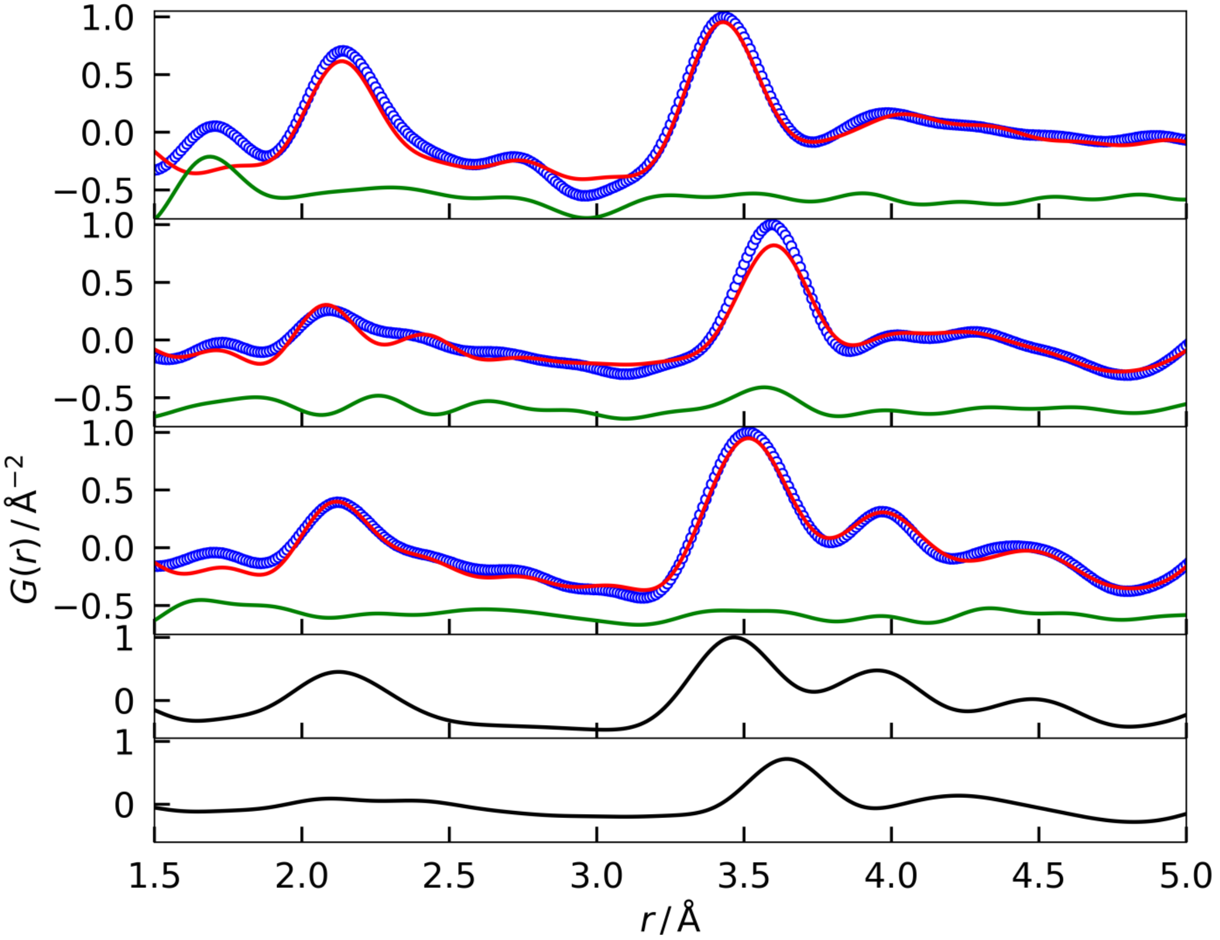

Figure S4: Zoom into Figure 4 of the main text to illustrate the local order of (from top to bottom) a spin-coated ZrO_2 film pre-annealed at 295 °C, two crystalline ZrO_2 films prepared from equally pre-annealed films and heated to 800 °C in a rapid thermal annealing process with a heating rate of ~100 K min^{-1}, and annealed at 900 °C in air at a slow heating rate of ~10 K min^{-1}, respectively (blue dots: data, red lines: calculated model, green line: difference curve, offset of -0.5 for clarity), along with the calculated reference PDFs from the ICSD database (references #658755 for monoclinic and #93124 for tetragonal with reduced values for U_{iso} extrapolated to room temperature).

Table S1: Q_{max} values applied in the Fourier transformation into the GIPDFs shown in the main text (with the exception of the calibration illustrated in Figure 3 where the Q_{max} values are included as a plot).

figure (text passage)	material	parameters	Q_{max} / Å^{-1}
Fig. 2 (Section 3.1)	Pt	3 nm thickness	17.2
	HfO_2	15 nm, crystalline	17.3
		30 nm, crystalline	21.1
		45 nm, crystalline	21.1
		15 nm, amorphous	12.5
		30 nm, amorphous	12.5
		45 nm, amorphous	14.4
Fig. 4 (Section 3.3)	ZrO_2	295 °C thermal treatment	21.0
		800 °C thermal treatment	21.0
		900 °C thermal treatment	21.0
Fig. 5 (Section 3.4)	Pt	normal incidence	17.0
		grazing incidence	24.0
